# Supplementary material for: Reintroduction of resistant frogs facilitates landscape-scale recovery in the presence of a lethal fungal disease
Source: Nat Commun. 2024 Nov 14;15:9436. doi: 10.1038/s41467-024-53608-4 (PMC11564713; doi:10.1038/s41467-024-53608-4)
Supplement: Supplementary file 1 — Supplementary Information [file 41467_2024_53608_MOESM1_ESM.pdf]

Supplementary Information for:

Reintroduction of resistant frogs facilitates landscape-scale recovery in the presence of a lethal fungal disease

Roland A. Knapp*, Mark Q. Wilber, Maxwell B. Joseph, Thomas C. Smith, & Robert L. Grasso

* **Author for correspondence:** roland.knapp@ucsb.edu

Supplementary Methods

Frog population recovery

Laboratory methods

Swab extracts were analyzed using standard Bd DNA extraction and qPCR methods[1], and extracts were analyzed singly instead of in triplicate[2]. For analysis of swabs collected during 2005–2014, we used standards developed from known concentrations of zoospores[1], and after 2014, we used standards based on single ITS1 PCR amplicons[3]. Based on paired comparisons between samples analyzed using both types of standards, Bd in the study area has an average of 60 ITS1 copies per zoospore. To express all qPCR results as the number of ITS1 copies, starting quantities obtained using the zoospore standard (measured as "zoospore equivalents") were multiplied by 60. In addition, all qPCR quantities (regardless of standard) were multiplied by 80 to account for the fact that DNA extracts from swabs were diluted 80-fold during extraction and PCR[4].

CMR model structure

We estimated survival and recruitment for each site using open population CMR models based on[5]. For each individual in a superpopulation of real and psuedo-individuals, $i = 1, ..., M$ on each survey $j = 1, ..., n_j$: $o_{i,j} = 1$ if the individual was not detected, and $o_{i,j} = 2$ if the individual was detected. Capture histories of M individuals are modeled, although only N_s individuals were captured. This parameter expanded data augmentation allows us to account for the possibility that undetected individuals may have recruited into the adult population[6]. Here, M was chosen to be three times the number of observed individuals ($3N_s$) to be considerably greater than our prior guess of N_s.

We denote the true state of individual i as $u_{i,t}$ for primary period $t = 1, ..., n_t$. The states that we consider are: $u_{i,t} = 1$ for individuals that have not recruited, $u_{i,t} = 2$ for live adults, and $u_{i,t} = 3$ for dead adults. Each survey $j = 1, ..., n_j$ occurs in one of the n_t primary periods, and we denote the primary period in which survey j takes place as t_j. Multiple primary periods can occur during a summer active season (see Methods). We set the year containing the first primary period to $y_{t=1} = 1$, and generally y_t represents the year containing primary period t. Years increment by one until the final year of the mark recapture efforts, which we denote n_y: $y \in \{1, 2, ..., n_y\}$. We assume that within a primary period, the state of each individual does not change (i.e., individuals do not recruit into the adult population, gain or lose Bd infection, or die). This assumption is justified by the short time intervals between surveys within primary periods, in cases where primary periods contain multiple secondary periods.

Live individuals are detected with probability p_j, which is modeled as:

$$p_j = \text{logit}^{-1}(X_j^{(p)} \beta^{(p)}), \tag{1}$$

where $X_j^{(p)}$ is a known row vector and $\beta^{(p)}$ an unknown parameter vector. Not recruited and dead individuals are never captured. We bundle these assumptions about the observation probabilities for survey j into an emission matrix Ω_j:

$$\Omega_j = \begin{array}{c} \\ \left(\begin{array}{cc} \text{Not detected} & \text{Detected} \\ 1 & 0 \\ 1 - p_j & p_j \\ 1 & 0 \end{array} \right) \end{array} \begin{array}{c} \\ \text{Not recruited} \\ \text{Alive} \\ \text{Dead} \end{array} \tag{2}$$

The state transition matrix $\Psi_{t,i}$ contains the probabilities of individual i transitioning from state $u_{i,t}$ (rows) to $u_{i,t+1}$ (columns) between primary period t and $t + 1$. For non-introduced (i.e., naturally recruited) individuals, this matrix is given by:

$$\Psi_{t,i} = \begin{array}{c} \\ \left(\begin{array}{ccc} \text{Not recruited} & \text{Alive} & \text{Dead} \\ 1 - \lambda_t & \lambda_t & 0 \\ 0 & \phi_t & 1 - \phi_t \\ 0 & 0 & 1 \end{array} \right) \end{array} \begin{array}{c} \\ \text{Not recruited} \\ \text{Alive} \\ \text{Dead} \end{array} \tag{3}$$

where λ_t is the probability of recruiting in time t and ϕ_t is the probability of survival in time t.

For introduced individuals, which have deterministic recruitment (i.e., they recruit when introduced), the state transition matrix is given by:

$$
\Psi_{t,i} = \begin{array}{c} \\ \end{array}
\begin{array}{ccc} \text{Not recruited} & \text{Alive} & \text{Dead} \end{array}
$$

$$
\Psi_{t,i} = \begin{pmatrix} 1 - I_{t,i} & I_{t,i} & 0 \\ 0 & \phi_t & 1 - \phi_t \\ 0 & 0 & 1 \end{pmatrix} \begin{array}{c} \text{Not recruited} \\ \text{Alive} \\ \text{Dead} \end{array} \tag{4}
$$

where $I_{t,i}$ is a known indicator function for whether individual i was introduced in primary period t.

We allow recruitment probabilities to vary in time via random effects, such that:

$$
\lambda_t = \text{logit}^{-1}(\alpha^{(\lambda)} + \epsilon_t^{(\lambda)}), \tag{5}
$$

where $\alpha^{(\lambda)}$ is an intercept parameter and $\epsilon_t^{(\lambda)}$ is an adjustment for time t.

Survival probabilities also vary in time, and as a function of known covariates:

$$
\phi_t = \text{logit}^{-1}(X_t^{(\phi)} \beta^{(\phi)} + \epsilon_t^{(\phi)}), \tag{6}
$$

where $X_t^{(\phi)}$ is a row vector of known covariates, $\beta^{(\phi)}$ is a column vector of unknown coefficients, and $\epsilon_t^{(\phi)}$ is an adjustment for time t.

To complete the specification of the Bayesian model, we specify priors for all unknown parameters. The recruitment parameter priors were specified as follows:

$$
\begin{aligned}
\alpha^{(\lambda)} &\sim N(0, 1) \\
\sigma^{(\lambda)} &\sim N_+(0, 1) \\
\epsilon_t^{(\lambda)} &\sim N(0, \sigma^{(\lambda)}),
\end{aligned} \tag{7}
$$

for periods $t = 1, ..., T$. Here N represents the normal distribution and N_+ the half normal distribution with positive support.

Survival parameter priors were specified similarly as:

$$
\begin{aligned}
\beta^{(\phi)} &\sim N(0, 1) \\
\sigma^{(\phi)} &\sim N_+(0, 1) \\
\epsilon_t^{(\phi)} &\sim N(0, \sigma^{(\phi)})
\end{aligned} \tag{8}
$$

for time $t = 1, ..., T$.

The detection model coefficient vector also received a standard normal prior $\beta^{(p)} \sim N(0, 1)$.

We computed the likelihood of each individual capture history using the forward algorithm, and we estimated the latent states using the forward-backward algorithm[5,7].

All of the code to specify and fit the model in Stan is available in the open source mrmr package[8].

The joint distribution of the resulting model can be written as follows:

$$[\alpha^{(\lambda)}, \sigma^{(\lambda)}, \epsilon^{(\lambda)}_{1:T}, \beta^{(\phi)}, \sigma^{(\phi)}, \epsilon^{(\phi)}_{1:T}, \beta^{(p)} \mid \mathbf{Y}] \propto \prod_{i=1}^{M} [Y_i \mid \alpha^{(\lambda)}, \epsilon^{(\lambda)}_{1:T}, \beta^{(\phi)}, \epsilon^{(\phi)}_{1:T}, \beta^{(p)}] \times \prod_{t=1}^{T} [\epsilon^{(\lambda)}_t \mid \sigma^{(\lambda)}][\epsilon^{(\phi])}_t | \sigma^{(\phi)}][\sigma^{(\lambda)}][\sigma^{(\phi)}][\alpha^{(\lambda)}][\beta^{(\phi)}][\beta^{(p)}], \tag{9}$$

where $\boldsymbol{Y}$ is an $M \times T$ detection matrix, and Y_i the capture history of individual i.

Among-site survival modeling

The objective of this analysis is to describe the influence of site, cohort, and individual level characteristics on post-translocation frog survival. By modeling survival estimates obtained from site-specific mrmr CMR analyses, we are in effect conducting an among-site meta-analysis. Although it would theoretically be possible to estimate survival covariate effects in a joint CMR model that integrates capture histories across all sites, this was impractical due to computational requirements of the CMR models (i.e., run time and memory).

We used Bayesian generalized linear mixed models to investigate predictors of survival among sites. The response y_i is binary, representing a point estimate of whether individual i survived in the year following translocation. We generated these point estimates by rounding the posterior median of 1-year post-introduction survival for each individual (from site-specific mrmr CMR models) and modeled the data using a Bernoulli distribution:

$$y_i \sim \text{Bernoulli}(p_i), \tag{10}$$

where p_i is the probability of survival.

We modeled variation in probabilities as follows:

$$\text{logit}(p_i) = \alpha + X_i\boldsymbol{\beta} + \boldsymbol{\nu}_{g[1:N]}, \tag{11}$$

where α is an intercept, X_i is a length K row vector of predictors, $\boldsymbol{\beta}$ is a column vector of predictor effects, and $\boldsymbol{\nu}$ a vector of group level random effects. Here $g[i]$ refers to the group g containing individual i, and we estimate an adjustment for each of the G groups $(\nu_1, ..., \nu_G)$.

These models were fit using the `stan_glmer()` function in the rstanarm package, with default priors described below[9]. These priors are vague, but include data-dependent scaling as follows to account for different input variable scales. However, because we standardized all predictor variables similarly to have equal variance (by centering and dividing by twice the sample standard deviation), the resulting priors are identical. Specifically, we have:

$$\begin{aligned} \alpha &\sim \text{Normal}(0, 2.5) \\ \beta_k &\sim \text{Normal}(0, 5), \end{aligned} \tag{12}$$

for $k = 1, ..., K$ where K is the number of predictor variables.

The default prior for group level adjustments $\nu_1, ..., \nu_G$ in rstanarm is a zero-mean Gaussian, where the covariance matrix is constructed from a correlation matrix with an LKJ prior, and a vector of variance parameters – the decomposition of variance prior with unit regularization, concentration, shape, and scale parameters[10].

We drew posterior samples using Dynamic Hamiltonian Monte Carlo in Stan, with four parallel chains, each run for 10,000 iterations, discarding the first half of each chain as warm-up draws[9]. We used Rhat statistics and trace plots to verify convergence. We considered models with different subsets of fixed and random effects, and used approximate leave-one-out cross validation to identify the best model[11].

Population viability modeling

Incorporating yearly variability in vital rates

We computed yearly survival probabilities for translocated adults σ_{A_T} and naturally recruited adults σ_{A_R} from the posterior distribution of individual state trajectories derived from mrmr CMR models. Although we observed yearly variability in adult survival within a population, the magnitude of this variability was small compared to among-population variability (Fig. 3). Thus, we did not include yearly within-population variability in adult survival in this analysis. However, within a population there was substantial yearly variability in the successful recruitment of adults, greater than what we would expect from Poisson variability around a mean value. Therefore, we allowed for yearly variability in the probability of year-1 juvenile survival (additional details provided in **Estimating model parameters** below). We also could have included environmental stochasticity in year-2 juvenile survival probability σ_{J_2}, but our elasticity and sensitivity analysis (see below, **Model analysis and simulation**) showed that this parameter had little effect on host growth rate relative to σ_{J_1} (Supplementary Fig. 4).

Estimating model parameters

The baseline parameter values for the model and how they were estimated are given in Supplementary Table 1. Parameters σ_{A_T} and σ_{A_R} were extracted directly from our CMR models (see **Supplementary Methods - Frog population recovery - Estimation of frog survival and abundance** and **CMR model structure** for details). For populations where we had a sufficient number of PIT-tagged, naturally-recruited adults, we observed that σ_{A_T} and σ_{A_R} could be notably different, with $\sigma_{A_R} > \sigma_{A_T}$ (Supplementary Fig. 3). For populations lacking sufficient numbers of naturally-recruited adults, we were unable to directly estimate σ_{A_R}, and instead set $\sigma_{A_R} = \sigma_{A_T}$.

To estimate the σ_{A_R}, we used the posterior distribution of predicted true states for naturally-recruited individuals (1=not recruited, 2=alive, 3=dead, as described in **Supplementary Methods - Frog population recovery - CMR model structure**), then calculated the posterior probability of individuals surviving between consecutive primary periods, conditional on being alive in the first primary period (e.g., given a value of 2 (alive) in the first primary period, how often was the value still 2 (alive) in the next primary period compared to 3 (dead)). This yielded posterior distributions for survival probabilities between primary periods. However, because the time interval between primary periods differed, the survival probabilities between different consecutive primary periods were not directly comparable. To address this, we converted the survival probabilities between each consecutive primary period to per day death rates, propagating the uncertainty from the posterior distributions. We then took a weighted average of these death rates, weighted by the time interval between primary periods, to get the average per day death rate over the entire CMR survey. We converted this per day death rate d back to a yearly survival probability using $\exp(-d \times 365 \text{ days})$. We used the same procedure for σ_{A_T} such that our estimates of average yearly survival probability were comparable between σ_{A_R} and σ_{A_T}.

Model analysis and simulation

We performed four analyses on our model. First, we considered a deterministic version of our model with no yearly heterogeneity in year-1 juvenile survival probability σ_{J_1}, and calculated the predicted long-run growth rate λ of a population for different values of σ_{A_R} and σ_{J_1}. We then fixed $\sigma_{J_1} = 0.1$ and calculated the predicted growth rate of our 12 populations.

Second, we performed a local elasticity and sensitivity analysis on λ with respect to parameters σ_{L_1}, σ_{L_2}, σ_{L_3}, σ_{J_1}, σ_{J_2}, σ_{A_R}, F, p_{L_2}, p_{J_1} to determine how small changes in these parameters could influence the long-run deterministic growth rate of populations (Supplementary Fig. 4). Note that λ is equally or more sensitive to changes in adult survival (σ_{A_R}) and survival of year-1 juveniles (σ_{J_1}) than it is to other model parameters, providing additional justification for focusing on variation in σ_{A_R} and σ_{J_1} in our viability analyses.

Third, we defined a version of the model with demographic and environmental stochasticity, where environmental stochasticity was represented by among-year variability in σ_{J_1}. We used

this model to simulate a one-time introduction of 40 translocated adult frogs. We ran this simulation 1000 times for each population and computed the probability of a population becoming extinct after 50 years given the observed parameter values and environmental stochasticity in σ_{J_1}. We varied the mean recruitment probability σ_{J_1} from 0 and 0.25 and drew values of σ_{J_1} each year from a beta distribution with a dispersion parameter of $\phi = 2$ (when $\sigma_{J_1} = 0.5$ and $\phi = 2$ the beta distribution is uniform between 0 and 1). Using different values of ϕ does not qualitatively change the existence of distinct extinction dynamics between populations with $\sigma_{A_R} < 0.5$ and those with $\sigma_{A_R} > 0.5$. However, increasing yearly variability in σ_{J_1} increases extinction risk for all populations. For example, if we set $\phi = 0.001$, such that in a given year essentially either all year-1 juveniles survive or all of them die, populations with $\sigma_{A_R} > 0.5$ need to have σ_{J_1} greater than 0.2 to have a 50-year extinction probability of less 50%. Because we do not PIT tag juveniles, we do not have CMR estimates for σ_{J_1} or ϕ. However, based on qualitative and semi-quantitative field observations over 25 years, a value of $\sigma_{J_1} = 0.25$ in the presence of Bd is probably a reasonable estimate for many populations. Thus, we expect our model predictions to be conservative with regards to population recovery.

Finally, we assessed whether our stochastic model could reproduce observed trajectories of population recovery. We focused on population 70550 because this was our longest CMR time series for a translocated population and because this population shows evidence of substantial post-translocation increases in adult abundance associated with population establishment and recovery. We simulated our model for 16 years, repeating the simulation 50,000 times. For each run and each year, we drew σ_{J_1} from a uniform distribution between 0 and 1 (or equivalently a beta distribution with mean 0.5 and $\phi = 2$). Using Approximate Bayesian Computing and rejection sampling[12], we identified the top 2% of trajectories (i.e., 1000 trajectories) that minimized the sum of squared errors between the observed and predicted data. The yearly σ_{J_1} values associated with these "best" trajectories represented an approximate posterior distribution[13]. Using these best fit trajectories, we assessed whether our model could qualitatively describe the patterns of recovery in the observed data for population 70550.

Supplementary References

1. Boyle, D., Boyle, D., Olsen, V., Morgan, J. & Hyatt, A. Rapid quantitative detection of chytridiomycosis (*Batrachochytrium dendrobatidis*) in amphibian samples using real-time Taqman PCR assay. *Diseases of Aquatic Organisms* **60**, 141–148 (2004).
2. Kriger, K., Hero, J. & Ashton, K. Cost efficiency in the detection of chytridiomycosis using PCR assay. *Diseases of Aquatic Organisms* **71**, 149–154 (2006).
3. Longo, A. V. *et al.* ITS1 copy number varies among *Batrachochytrium dendrobatidis* strains: Implications for qPCR estimates of infection intensity from field-collected amphibian skin swabs. *PLoS ONE* **8**, e59499 (2013).
4. Vredenburg, V. T., Knapp, R. A., Tunstall, T. S. & Briggs, C. J. Dynamics of an emerging disease drive large-scale amphibian population extinctions. *Proceedings of the National Academy of Sciences USA* **107**, 9689–9694 (2010).

5. Joseph, M. B. & Knapp, R. A. Disease and climate effects on individuals drive post-reintroduction population dynamics of an endangered amphibian. *Ecosphere* **9**, e02499 (2018).
6. Royle, J. A. & Dorazio, R. M. Parameter-expanded data augmentation for Bayesian analysis of capture–recapture models. *Journal of Ornithology* **152**, 521–537 (2012).
7. Zucchini, W. & MacDonald, I. L. *Hidden Markov Models for Time Series: An Introduction Using R.* (Chapman and Hall/CRC, 2009). doi:10.1201/9781420010893.
8. Joseph, M. B. mrmr: mark recapture miscellany in R. (2019).
9. Goodrich, B., Gabry, J., Ali, I. & Brilleman, S. rstanarm: Bayesian applied regression modeling via Stan. (2022).
10. Lewandowski, D., Kurowicka, D. & Joe, H. Generating random correlation matrices based on vines and extended onion method. *Journal of Multivariate Analysis* **100**, 1989–2001 (2009).
11. Vehtari, A., Gelman, A. & Gabry, J. Practical Bayesian model evaluation using leave-one-out cross-validation and WAIC. *Statistics and Computing* **27**, 1413–1432 (2016).
12. Kosmala, M. *et al.* Estimating wildlife disease dynamics in complex systems using an Approximate Bayesian Computation framework. *Ecological Applications* **26**, 295–308 (2016).
13. Beaumont, M. A. Approximate Bayesian Computation in evolution and ecology. *Annual Review of Ecology, Evolution, and Systematics* **41**, 379–406 (2010).
14. Briggs, C. J., Knapp, R. A. & Vredenburg, V. T. Enzootic and epizootic dynamics of the chytrid fungal pathogen of amphibians. *Proceedings of the National Academy of Sciences USA* **107**, 9695–9700 (2010).

Supplementary Figures

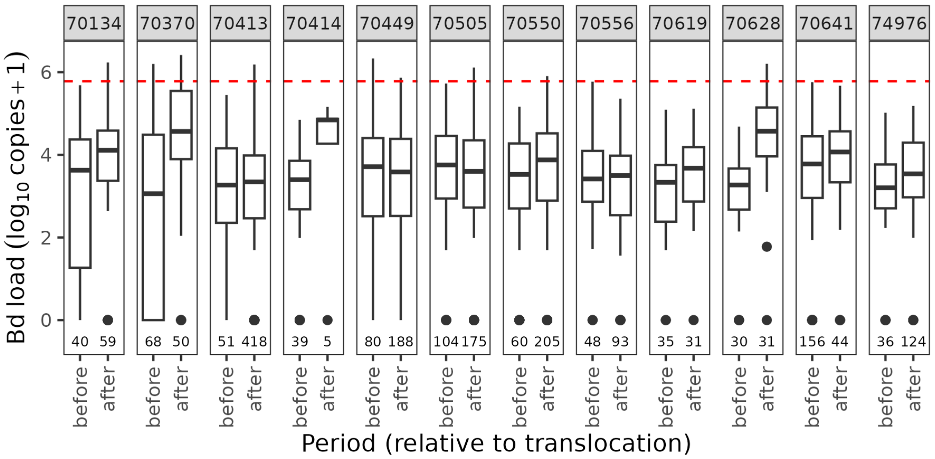

Supplementary Fig. 1. Bd loads on frogs before and after translocation. The "before" period is immediately prior to translocation and the "after" period is the 1-year period after translocation. Bd loads are expressed as the number of ITS1 copies per skin swab, as estimated by qPCR of the Bd ITS1 region. Box plots show medians (horizontal line), first and third quartiles (hinges), largest and smallest values within 1.5x interquartile range (whiskers), and values outside the 1.5x interquartile range (dots). Sample sizes are provided immediately above the x-axis. The red dashed horizontal line indicates the approximate load above which severe disease is typically observed[4]. This threshold (5.8 ITS1 copies - on a log10 scale) is commonly exceeded during Bd epizootics, but is exceeded only rarely in recovering populations[14]. Source data are provided as a Source Data file.

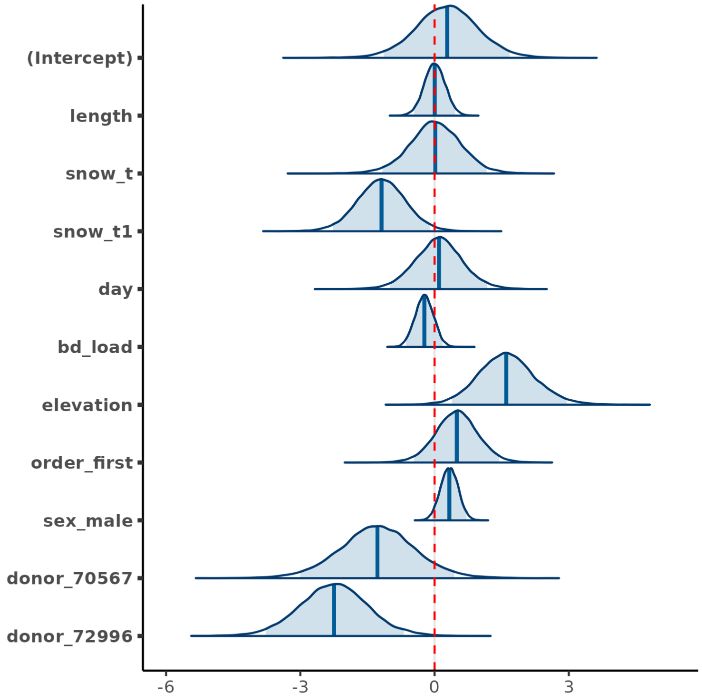

Supplementary Fig. 2. Predictors of post-translocation frog survival in the among-site meta-analysis. Depicted distributions for the intercept and all predictor variables are the estimated posterior density curves and shaded 95% uncertainty intervals from the best model. In the Bayesian framework in which the model was developed, variables are considered important predictors if the associated uncertainty interval does not overlap zero (indicated by the dashed red line). Predictor variables shown on the y-axis are defined as follows: length = frog size, snow_t = winter severity in the year of translocation (measured on April 1), snow_t1 = winter severity in the year following translocation (measured on April 1), day = day of year on which a translocation was conducted, bd_load = Bd load, elevation = site elevation, order_first = within-site translocation order, sex_male = frog sex, donor_70567 and donor_72996 = donor population. Note that Bd load is not an important predictor of post-translocation frog survival.

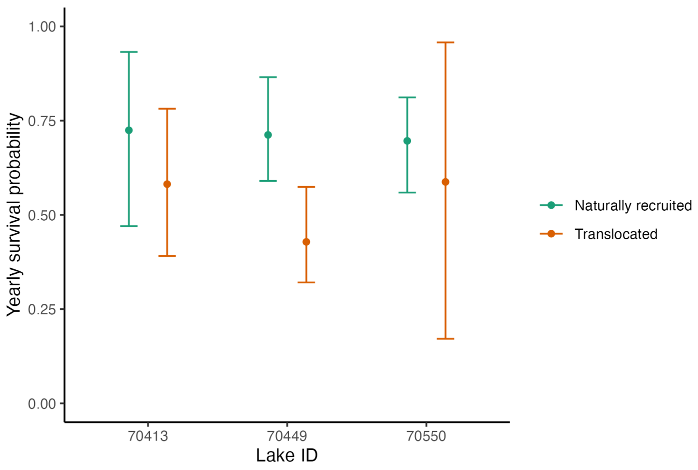

Supplementary Fig. 3. Average yearly survival probabilities of translocated versus naturally recruited adults. Naturally recruited individuals were the result of reproduction by translocated frogs at each site. In contrast to Fig. 3, these are not survival probabilities from the first year following translocation, but instead represent averaged survival probabilities across multiple years and cohorts. Points are median estimates and error bars give the 95% uncertainty intervals around the estimates, accounting for yearly variation in survival probabilities. All estimates were derived using the mrmr package, and the methods for calculation are described in Supplemenary Information - Population viability modeling - Estimating model parameters. Source data are provided as a Source Data file.

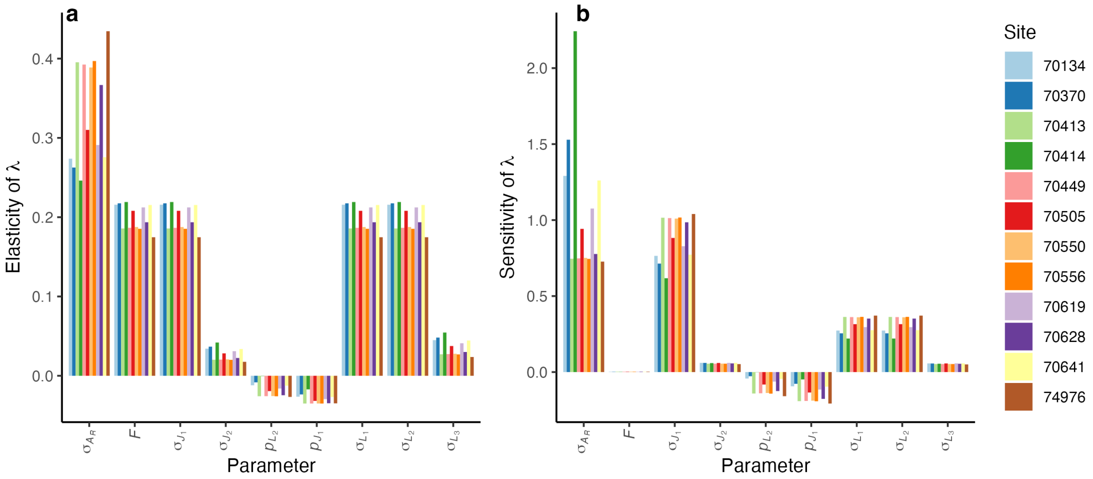

Supplementary Fig. 4. Sensitivity analysis of the stage-structured *R. sierrae* model. **a** Elasticity of λ with changes in nine parameters: σ_{A_R} (yearly survival probability of naturally recruited adults), F (number of eggs produced by a female frog in a year that successfully hatch), σ_{J_1} (yearly probability of survival of year-1 juveniles that also affects recruitment), σ_{J_2} (yearly probability of survival of year-2 juveniles), p_L_2 (probability that year-2 tadpoles metamorph into juveniles), p_{J_1} (probability that year-1 juveniles mature to adults), σ_{L_1} (survival probability of year-1 tadpoles), σ_{L_2} (survival probability of year-2 tadpoles), and σ_{L_3} (survival probability of year-3 tadpoles). **b** Sensitivity of λ with changes in the same nine parameters. Note that F has different units than the other parameters, which is why its sensitivity is low. Sensitivities of all other parameters are comparable. Elasticity and sensitivity are calculated at the default parameter values for each population (Supplementary Table 1). We did not include p_{L_1} in this analysis because $p_{L_1} = 1$ for all populations in this study – tadpoles spend at least a year as tadpoles before metamorphosing (Supplementary Table 1).

Supplementary Tables

Supplementary Table 1. Description and values of parameters used in the population viability model.

Parameter[a]	Value	Source
σ_{L_1}, Yearly survival probability of year-1 tadpoles	0.7	Estimated from field data, observations
σ_{L_2}, Yearly survival probability of year-2 tadpoles	0.7	Estimated from field data, observations
σ_{L_3}, Yearly survival probability of year-3 tadpoles	0.7	Estimated from field data, observations
σ_{J_1}, Yearly survival probability of year-1 juveniles.	Varies yearly	Varies. Bounds estimated from field data, observations
σ_{J_2}, Yearly survival probability of year-2 juveniles	0.5	Estimated from field data, observations
σ_{A_R}, Yearly survival probability of naturally recruited adults	Varies by population	Estimated from CMR studies
σ_{A_T}, Yearly survival probability of translocated adults	Varies by population	Estimated from CMR studies
p_{L_1}, Probability of a year-1 tadpoles remaining as a tadpoles	1	Estimated from field data, observations
p_{L_2}, Probability of a year-2 tadpoles remaining as a tadpoles	0.25	Estimated from field data, observations
p_{J_1}, Probability of a year-1 juvenile remaining as a juvenile	0.25	Estimated from field data, observations
p_F, Probability of an adult female reproducing in a year	0.5	Could be as high at 1, based on field observations
ρ, Proportion of adults that are female	0.5	Estimated from field data
F, Number of surviving eggs produced by an adult female	100	From observations of captive frogs

[a]All survival probabilities are in the presence of the fungal pathogen Bd.
