# Supplementary material for: Reintroduction of resistant frogs facilitates landscape-scale recovery in the presence of a lethal fungal disease
Source: Nat Commun. 2024 Nov 14;15:9436. doi: 10.1038/s41467-024-53608-4 (PMC11564713; doi:10.1038/s41467-024-53608-4)
Supplement: Supplementary file 3 — Reporting Summary [file 41467_2024_53608_MOESM3_ESM.pdf]

nature portfolio

Corresponding author(s): Roland A. Knapp

Last updated by author(s): Aug 5, 2024

Reporting Summary

Nature Portfolio wishes to improve the reproducibility of the work that we publish. This form provides structure for consistency and transparency in reporting. For further information on Nature Portfolio policies, see our Editorial Policies and the Editorial Policy Checklist.

Statistics

For all statistical analyses, confirm that the following items are present in the figure legend, table legend, main text, or Methods section.

n/a	Confirmed	
☐	☒	The exact sample size (*n*) for each experimental group/condition, given as a discrete number and unit of measurement
☐	☒	A statement on whether measurements were taken from distinct samples or whether the same sample was measured repeatedly
☒	☐	The statistical test(s) used AND whether they are one- or two-sided<br>*Only common tests should be described solely by name; describe more complex techniques in the Methods section.*
☐	☒	A description of all covariates tested
☒	☐	A description of any assumptions or corrections, such as tests of normality and adjustment for multiple comparisons
☐	☒	A full description of the statistical parameters including central tendency (e.g. means) or other basic estimates (e.g. regression coefficient) AND variation (e.g. standard deviation) or associated estimates of uncertainty (e.g. confidence intervals)
☒	☐	For null hypothesis testing, the test statistic (e.g. *F*, *t*, *r*) with confidence intervals, effect sizes, degrees of freedom and *P* value noted<br>*Give P values as exact values whenever suitable.*
☐	☒	For Bayesian analysis, information on the choice of priors and Markov chain Monte Carlo settings
☐	☒	For hierarchical and complex designs, identification of the appropriate level for tests and full reporting of outcomes
☒	☐	Estimates of effect sizes (e.g. Cohen's *d*, Pearson's *r*), indicating how they were calculated

Our web collection on statistics for biologists contains articles on many of the points above.

Software and code

Policy information about availability of computer code

Data collection	Field data were collected using the Fulcrum app and custom forms. The current version is v. 6.9, but earlier versions were also used during the data collection period.
Data analysis	Data were analyzed using R Statistical Software (v. 4.4.4). As stated in the "Code availability" section of our manuscript, "R code to replicate our analyses is available at https://github.com/SNARL1/translocation. R code to analyze the CMR datasets is available at https://github.com/SNARL1/cmr-analysis."

For manuscripts utilizing custom algorithms or software that are central to the research but not yet described in published literature, software must be made available to editors and reviewers. We strongly encourage code deposition in a community repository (e.g. GitHub). See the Nature Portfolio guidelines for submitting code & software for further information.

Data

Policy information about availability of data

All manuscripts must include a data availability statement. This statement should provide the following information, where applicable:

- Accession codes, unique identifiers, or web links for publicly available datasets
- A description of any restrictions on data availability
- For clinical datasets or third party data, please ensure that the statement adheres to our policy

As stated in the "Data availability" section of our manuscript, "The data used in this study are available at https://github.com/SNARL1/translocation. The CMR datasets from each reintroduction site are available at https://github.com/SNARL1/cmr-analysis."

Research involving human participants, their data, or biological material

Policy information about studies with human participants or human data. See also policy information about sex, gender (identity/presentation), and sexual orientation and race, ethnicity and racism.

Reporting on sex and gender	NA
Reporting on race, ethnicity, or other socially relevant groupings	NA
Population characteristics	NA
Recruitment	NA
Ethics oversight	NA

Note that full information on the approval of the study protocol must also be provided in the manuscript.

Field-specific reporting

Please select the one below that is the best fit for your research. If you are not sure, read the appropriate sections before making your selection.

☐ Life sciences ☐ Behavioural & social sciences ☒ Ecological, evolutionary & environmental sciences

For a reference copy of the document with all sections, see nature.com/documents/nr-reporting-summary-flat.pdf

Life sciences study design

All studies must disclose on these points even when the disclosure is negative.

Sample size	NA
Data exclusions	NA
Replication	NA
Randomization	NA
Blinding	NA

Behavioural & social sciences study design

All studies must disclose on these points even when the disclosure is negative.

Study description	NA
Research sample	NA
Sampling strategy	NA
Data collection	NA

Timing	NA
Data exclusions	NA
Non-participation	NA
Randomization	NA

Ecological, evolutionary & environmental sciences study design

All studies must disclose on these points even when the disclosure is negative.

Study description	Our study including three main analyses: (1) descriptive statistics of reproduction and recruitment in reintroduced frog populations, (2) a meta-analysis to identify important predictors of survival of reintroduced frogs, and (3) a viability model to provide insights into long-run population growth rates and viability. Only the analysis in (2) is associated with treatment factors, design structure, experimental units, etc. To accommodate the hierarchical structure of the dataset, in which translocated cohorts of frogs are nested within reintroduction sites, and individual frogs are nested within translocated cohorts, we used multilevel Bayesian models. Included predictor variables describe characteristics of sites, translocated cohorts, and individuals (Bd load, sex, frog size, site elevation, winter severity in the year of translocation, winter severity in the year following translocation, donor population, day of year on which a translocation was conducted, and translocation order). We used 1-year post-translocation survival estimates from capture-mark-recapture (CMR) models as the response. Estimated survival was rounded to integer values to produce a binary outcome, and modeled with a Bernoulli distribution. Group-level (random) effects included site_id, translocation_id, or translocation_id nested within site_id.
Research sample	The research sample is a group of Sierra Nevada yellow-legged frogs (Rana sierrae) collected from three donor sites in Yosemite National Park and translocated to 12 nearby lakes. Both males and females were represented and females were slightly overrepresented.
Sampling strategy	The sampling strategy to estimate frog survival, recruitment, and adult population size was a CMR robust design in which as many frogs as possible were captured from each reintroduced population as several consecutive days. Site-specific estimates of frog survival, recruitment, and adult population size were derived from a site-specific Bayesian open-population Jolly-Seber CMR model with known additions to the population (i.e., translocated cohorts).
Data collection	Data were collected by R. Knapp and T. Smith, with the assistance of numerous field technicians. Data were collected on smartphones using the Fulcrum app.
Timing and spatial scale	CMR data were collected between 2006 and 2022, producing 2-16 years of data for each reintroduced population. In each year, CMR surveys were conducted approximately once per month during the summer active season (generally mid-June to late-August). Data were collected from each of the 12 reintroduced populations in Yosemite National Park.
Data exclusions	No data were excluded from the analyses.
Reproducibility	We conducted a total of 24 translocations to the 12 recipient sites (1-4 translocations per recipient site). All reintroduction attempts were successful.
Randomization	Frogs in each of the 24 translocated cohorts were collected from one of three donor sites. Collected individuals were those detected during shoreline surveys, with no attempt at randomization. Randomization was not logistically feasible nor is the associated additional handling of frogs justifiable for this endangered species.
Blinding	Blinding during data collection is not possible because every data collector knows the site at which they are working. In addition, blinding was not used during data analysis because the data were analyzed using a model, and blinding would therefore serve no purpose.

Did the study involve field work? ☒ Yes ☐ No

Field work, collection and transport

Field conditions	Field conditions during CMR surveys spanned a wide range of temperature and weather. Typical temperatures ranged from approximately 10 to 24 C, and weather ranged from sunny to cloudy. CMR was halted during periods of rain.
Location	The study was conducted in Yosemite National Park, California, USA. The centroid of all reintroduction sites is approximately lat 37.9780, lon -119.1561. Elevations ranged from 2471 to 3505 m. Water depths of the reintroduction lakes ranged from 4 to 25 m.
Access & import/export	All data collection protocols were in accordance with permits provided by the US Fish and Wildlife Service (Permit # TE-40090B: 2014, 2016, 2019), Yosemite National Park (2006-2022), and University of California - Santa Barbara Institutional Animal Care and Use Committee (2006-2022).

Disturbance	Disturbance caused by the study was limited to that associated with CMR surveys, and included temporary stress of captured frogs, slight trampling of shoreline vegetation, and camping-related impacts. All were minimized as per the conditions in our research permits and through the use of Leave No Trace practices.

Reporting for specific materials, systems and methods

We require information from authors about some types of materials, experimental systems and methods used in many studies. Here, indicate whether each material, system or method listed is relevant to your study. If you are not sure if a list item applies to your research, read the appropriate section before selecting a response.

Materials & experimental systems

n/a	Involved in the study	
☒	☐	Antibodies
☒	☐	Eukaryotic cell lines
☒	☐	Palaeontology and archaeology
☐	☒	Animals and other organisms
☒	☐	Clinical data
☒	☐	Dual use research of concern
☒	☐	Plants

Methods

n/a	Involved in the study	
☒	☐	ChIP-seq
☒	☐	Flow cytometry
☒	☐	MRI-based neuroimaging

Antibodies

Antibodies used	NA
Validation	NA

Eukaryotic cell lines

Policy information about cell lines and Sex and Gender in Research

Cell line source(s)	NA
Authentication	NA
Mycoplasma contamination	NA
Commonly misidentified lines (See ICLAC register)	NA

Palaeontology and Archaeology

Specimen provenance	NA
Specimen deposition	NA
Dating methods	NA

☐ Tick this box to confirm that the raw and calibrated dates are available in the paper or in Supplementary Information.

Ethics oversight	NA

Note that full information on the approval of the study protocol must also be provided in the manuscript.

Animals and other research organisms

Policy information about studies involving animals; ARRIVE guidelines recommended for reporting animal research, and Sex and Gender in Research

Laboratory animals	NA
Wild animals	Animals captured as part of this research were Sierra Nevada yellow-legged frogs (Rana sierrae), and included adults, juveniles, and tadpoles. Frogs were captured using hand nets. For translocations, adult frogs were transported on foot or via helicopter. At the conclusion of the study, any of the original translocated frogs that were still alive were allowed to remain at the reintroduction sites

	where they will continue contributing to the establishment and growth of the reintroduced populations. Ages of translocated frogs are unknown, but are assumed to be 6-10 years.
Reporting on sex	Every captured adult frog was sexed based on the presence/absence of male secondary sexual characteristics (i.e., nuptial pads on thumbs). Sex was included as a predictor variable in the meta-analysis. A total of 428 females and 351 males were included.
Field-collected samples	The study did not involve live animal samples collected from the field. The only samples were frog skin swabs analyzed using qPCR methods.
Ethics oversight	All data collection protocols were in accordance with permits provided by the US Fish and Wildlife Service (Permit # TE-40090B: 2014, 2016, 2019), Yosemite National Park (2006-2022), and University of California - Santa Barbara Institutional Animal Care and Use Committee (2006-2022).

Note that full information on the approval of the study protocol must also be provided in the manuscript.

Clinical data

Policy information about clinical studies

All manuscripts should comply with the ICMJEguidelines for publication of clinical research and a completedCONSORT checklist must be included with all submissions.

Clinical trial registration	NA
Study protocol	NA
Data collection	NA
Outcomes	NA

Dual use research of concern

Policy information about dual use research of concern

Hazards

Could the accidental, deliberate or reckless misuse of agents or technologies generated in the work, or the application of information presented in the manuscript, pose a threat to:

No	Yes	
☒	☐	Public health
☒	☐	National security
☒	☐	Crops and/or livestock
☒	☐	Ecosystems
☒	☐	Any other significant area

Experiments of concern

Does the work involve any of these experiments of concern:

No	Yes	
☒	☐	Demonstrate how to render a vaccine ineffective
☒	☐	Confer resistance to therapeutically useful antibiotics or antiviral agents
☒	☐	Enhance the virulence of a pathogen or render a nonpathogen virulent
☒	☐	Increase transmissibility of a pathogen
☒	☐	Alter the host range of a pathogen
☒	☐	Enable evasion of diagnostic/detection modalities
☒	☐	Enable the weaponization of a biological agent or toxin
☒	☐	Any other potentially harmful combination of experiments and agents

Plants

Seed stocks	NA
Novel plant genotypes	NA
Authentication	NA

ChIP-seq

Data deposition

- [ ] Confirm that both raw and final processed data have been deposited in a public database such as GEO.
- [ ] Confirm that you have deposited or provided access to graph files (e.g. BED files) for the called peaks.

Data access links<br>*May remain private before publication.*	NA
Files in database submission	NA
Genome browser session<br>(e.g. UCSC)	NA

Methodology

Replicates	NA
Sequencing depth	NA
Antibodies	NA
Peak calling parameters	NA
Data quality	NA
Software	NA

Flow Cytometry

Plots

Confirm that:

- [ ] The axis labels state the marker and fluorochrome used (e.g. CD4-FITC).
- [ ] The axis scales are clearly visible. Include numbers along axes only for bottom left plot of group (a 'group' is an analysis of identical markers).
- [ ] All plots are contour plots with outliers or pseudocolor plots.
- [ ] A numerical value for number of cells or percentage (with statistics) is provided.

Methodology

Sample preparation	NA
Instrument	NA
Software	NA
Cell population abundance	NA

Gating strategy: NA

☐ Tick this box to confirm that a figure exemplifying the gating strategy is provided in the Supplementary Information.

Magnetic resonance imaging

Experimental design

Design type	NA
Design specifications	NA
Behavioral performance measures	NA

Acquisition

Imaging type(s)	NA
Field strength	NA
Sequence & imaging parameters	NA
Area of acquisition	NA

Diffusion MRI ☐ Used ☒ Not used

Preprocessing

Preprocessing software	NA
Normalization	NA
Normalization template	NA
Noise and artifact removal	NA
Volume censoring	NA

Statistical modeling & inference

Model type and settings	NA
Effect(s) tested	NA

Specify type of analysis: ☐ Whole brain ☐ ROI-based ☐ Both

Statistic type for inference (See Eklund et al. 2016)	NA
Correction	NA

Models & analysis

n/a	Involved in the study
☒	☐ Functional and/or effective connectivity
☒	☐ Graph analysis
☒	☐ Multivariate modeling or predictive analysis

Functional and/or effective connectivity	NA
Graph analysis	NA
Multivariate modeling and predictive analysis	NA
